# Noninvasive monitoring of PaCO_2_ during one-lung ventilation and minimal access surgery in adults: End-tidal versus transcutaneous techniques

**DOI:** 10.4103/0972-9941.30680

**Published:** 2007

**Authors:** Paul Cox, Joseph D Tobias

**Affiliations:** University of Missouri School of Medicine, Columbia, Missouri, USA; * Department of Anesthesiology and Pediatrics, University of Missouri, Columbia, Missouri, USA

**Keywords:** End-tidal CO_2_, thoracoscopy, transcutaneous CO_2_

## Abstract

**Background:**

Previous studies have suggested that end-tidal CO_2_ (ET-CO_2_) may be inaccurate during one-lung ventilation (OLV). This study was performed to compare the accuracy of the noninvasive monitoring of PCO_2_ using transcutaneous CO_2_ (TC-CO_2_) with ET-CO_2_ in patients undergoing video-assisted thoracoscopic surgery (VATS) during OLV.

**Materials and Methods:**

In adult patients undergoing thoracoscopic surgical procedures, PCO_2_ was simultaneously measured with TC-CO_2_ and ET-CO_2_ devices and compared with PaCO_2_.

**Results:**

The cohort for the study included 15 patients ranging in age from 19 to 71 years and in weight from 76 to 126 kg. During TLV, the difference between the TC-CO_2_ and the PaCO_2_ was 3.0 ± 1.8 mmHg and the difference between the ET-CO_2_ and PaCO_2_ was 6.2 ± 4.7 mmHg (*P*=0.02). Linear regression analysis of TC-CO2 *vs.* PaCO_2_ resulted in an r^2^ = 0.6280 and a slope = 0.7650 ± 0.1428, while linear regression analysis of ET-CO_2_ *vs.* PaCO_2_ resulted in an r^2^ = 0.05528 and a slope = 0.1986 ± 0.1883. During OLV, the difference between the TC-CO_2_ and PaCO_2_ was 3.5 ± 1.7 mmHg and the ET-CO_2_ to PaCO_2_ difference was 9.6 ± 3.6 mmHg (*P*=0.03 *vs.* ET-CO_2_ to PaCO_2_ difference during TLV; and *P*<0.0001 *vs.* TC-CO_2_ to PaCO_2_ difference during OLV). In 13 of the 15 patients, the TC-CO_2_ value was closer to the actual PaCO_2_ than the ET-CO_2_ value (*P* =0.0001). Linear regression analysis of TC-CO_2_ *vs.* PaCO_2_ resulted in an r^2^ = 0.7827 and a slope = 0.8142 ± 0.0.07965, while linear regression analysis of ET-CO_2_ *vs.* PaCO_2_ resulted in an r^2^ = 0.2989 and a slope = 0.3026 ± 0.08605.

**Conclusions:**

During OLV, TC-CO_2_ monitoring provides a better estimate of PaCO_2_ than ET-CO2 in patients undergoing VATS.

The measurement of the partial pressure of carbon dioxide in the arterial blood (PaCO_2_) is used to determine the adequacy of ventilation and to guide changes in mechanical ventilation. Although arterial sampling provides this information, frequent arterial blood gas (ABG) analysis is expensive, time restrictive and provides only intermittent data for what may be a rapidly changing value. To overcome such problems, noninvasive monitors are frequently used to provide a continuous estimate of the PaCO_2_. In the operating room, end-tidal (ET) CO_2_ monitoring remains the standard of care for continuous and noninvasive PaCO_2_ monitoring. However, various factors, including patient positioning, sampling errors and ventilation-perfusion mismatch due to dead space or shunt, may significantly affect the arterial to ET-CO_2_ gradient.[1–3]

One-lung ventilation (OLV) is commonly used during thoracic surgery to allow surgical access to the operative lung and to eliminate lung movement during ventilation. The technique is helpful during open thoracic procedures and a necessity for minimal access thoracoscopic procedures. During OLV, the bronchus of the affected lung is occluded by a bronchial blocker or isolated by a double-lumen endotracheal tube, with oxygenation and ventilation supported by the nonoperative lung. Even with effective hypoxic pulmonary vasoconstriction, the technique results in an increase in the shunt fraction and perfusion of the nonventilated lung. Previous studies have demonstrated a significant arterial to ET-CO_2_ gradient during one-lung OLV.[4–6] The efficacy of ET-CO_2_ monitoring is limited during such procedures, suggesting that alternative noninvasive monitors of PaCO_2_ may be needed. Although used predominantly in the neonatal and pediatric population, there is increasing interest in the use of and recent reports of transcutaneous (TC) CO_2_ monitoring in the adult population.[5–9] The current study prospectively compares ET-CO_2_ and TC-CO_2_ monitoring during OLV in adults undergoing video-assisted thoracoscopic surgery.

## MATERIALS AND METHODS

The study was approval by the Institutional Review Board and the Committee for the Protection of Human Subjects of the University of Missouri. Verbal informed consent was obtained from each patient. The patient population included patients scheduled for minimal access thoracic surgery and OLV, who were 18 years of age or older and in whom intraarterial access was deemed necessary. OLV was provided by either a bronchial blocker or a double lumen endotracheal tube. Effective lung separation was confirmed by clinical auscultation and fiberoptic bronchoscopy prior to the start of the procedure. Intrathoracic CO_2_ insufflation was not used during the procedure.

### Transcutaneous and end-tidal carbon dioxide monitoring

ET-CO_2_ was measured using an infrared analyzer with a side stream sampler attached at the elbow between the endotracheal tube and the anesthesia circuit. Prior to use, the ET-CO_2_ device was calibrated according to the manufacturer's recommendations. TC-CO_2_ was measured with a commercially available TC-CO_2_ device (Sentec AG, Therwil, Switzerland). This device is based on a Stow-Severinghaus-type CO_2_ sensor combined with a pulse oximeter and is attached to the patient's earlobe by a low pressure attachment clip.[10,11] The *in vitro* 90% response time is <50s for the CO_2_ electrode. Prior to placement, the sensor was prepared and calibrated according to the manufacturer's recommendations. The sensor is calibrated *in vitro* by using a one-point dry gas calibration with 7% carbon dioxide. The working temperature of the sensor is 42°C. The sensor was cleaned with alcohol and dried before application. One drop of contact gel was applied to the center of the sensor prior to placement on the patient's earlobe. Oxygen saturation values are available immediately, while TC-CO_2_ values are available after a 2-3 min calibration time.

### Data collection and statistical analysis

ABG analyses were obtained following endotracheal intubation during two-lung ventilation (TLV) and as clinically indicated during OLV. When an ABG was obtained, the ET-CO_2_ and TC-CO_2_ were simultaneously recorded on a data sheet. Calculation of the absolute difference between the noninvasive monitor (ET-CO_2_ or TC-CO_2_) and the PaCO_2_ was performed. Negative numbers were not used because this could artificially lower the mathematical mean of the differences between the noninvasive monitors of CO_2_ and the PaCO_2_. If multiple ABGs were obtained during OLV, the absolute differences between the PaCO_2_ and the noninvasive monitors were averaged and counted as a single data point. This was done to avoid biasing the data by overrepresentation of any one patient as the number of ABGs varied for each patient. The absolute difference between the ET-CO_2_ and PaCO_2_ was compared to the absolute difference between the TC-CO_2_ and the PaCO_2_ during TLV and OLV using a non-paired t-test. A contingency table with a Fisher's exact test was used to compare the times that each of the noninvasive monitors was closest to the actual PaCO_2_. Using the raw numbers from all of the individual sample sets (PaCO_2_/ET-CO_2_/TC-CO_2_) obtained during TLV and OLV, linear regression analysis and Bland-Altman analyses were performed.

## RESULTS

**Figure 1:**
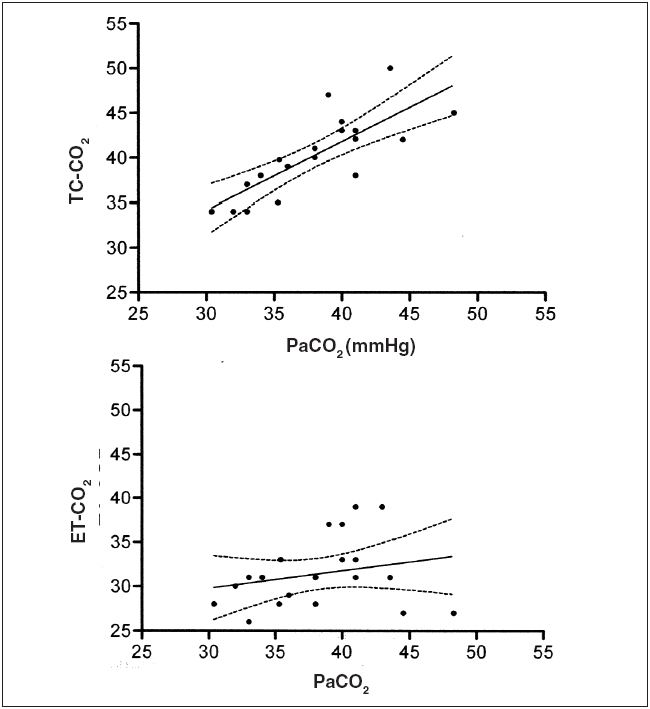
Linear regression analysis during two-lung ventilation of TCCO_2_ versus PaCO_2_ (above) and ET-CO_2_ vs. PaCO_2_ (below). Analysis of TC-CO_2_ vs. PaCO_2_ revealed r^2^ = 0.6280 and slope = 0.7650 ± 0.1428 (95% confidence intervals: 0.4367 to 1.066). Analysis of ET-CO_2_ vs. PaCO_2_ revealed of = r^2^ = 0.05528 and slope = 0.1986 ± 0.1883 (95% confidence intervals: −0.1956 to 0.5928)

The cohort for the study included 15 patients ranging in age from 19 to 71 years (46.9 ± 17.3 years) and in weight from 76 to 126 kg (93.4 ± 12.4 kg). There were 11 men and 4 women. Nineteen sample sets (PaCO_2_/ET-CO_2_/TC-CO_2_) were obtained during TLV and 27 were obtained during OLV. During TLV, the difference between the TC-CO_2_ and the PaCO_2_ was 3.0 ± 1.8 mmHg with a range of 0 to 8 mmHg and the difference between the ET-CO_2_ and the PaCO_2_ was 6.2 ± 4.7 mmHg with a range of 2 to 18 mmHg (*P*=0.02 *vs.* TC-CO_2_ to PaCO_2_ difference) [Table 1]. During TLV, the TC-CO_2_ value was closer to the actual PaCO_2_ in 10 patients, the ET-CO_2_ value was closer in 2 patients and there was no difference in 3 patients (*P*=NS). During TLV, linear regression analysis of TC-CO_2_ *vs.* PaCO_2_ resulted in an r^2^ = 0.6280 and a slope = 0.7650 ± 0.1428 (95% confidence intervals: 0.4367 to 1.066). During TLV, linear regression analysis of ET-CO_2_ *vs.* PaCO_2_ resulted in an r^2^ = 0.05528 and a slope = 0.1986 ± 0.1883 (95% confidence intervals: -0.1956 to 0.5928) [Figure 1]. During TLV, Bland-Altman analysis of TC *vs.* PaCO_2_ revealed a bias of +2.2 mmHg and a precision of ± 3.0 mmHg and analysis of ET *vs.* PaCO_2_ revealed a bias of -7.3 mmHg and a precision of ± 5.5 mmHg.

**Table 1:** End-tidal and transcutaneous differences *vs.* PaCO_2_ during one-lung ventilation and two-lung ventilation

	*TC-CO_2_ to PaCO_2_ difference (mmHg)*	*ET-CO_2_ to PaCO_2_ difference (mmHg)*	*P value*
*Two-lung ventilation*	3.0 ± 1.8	6.2 ± 4.7	0.02
*One-lung ventilation*	3.5 ± 1.7	9.6 ± 3.6	*P*<0.001

**Figure 2:**
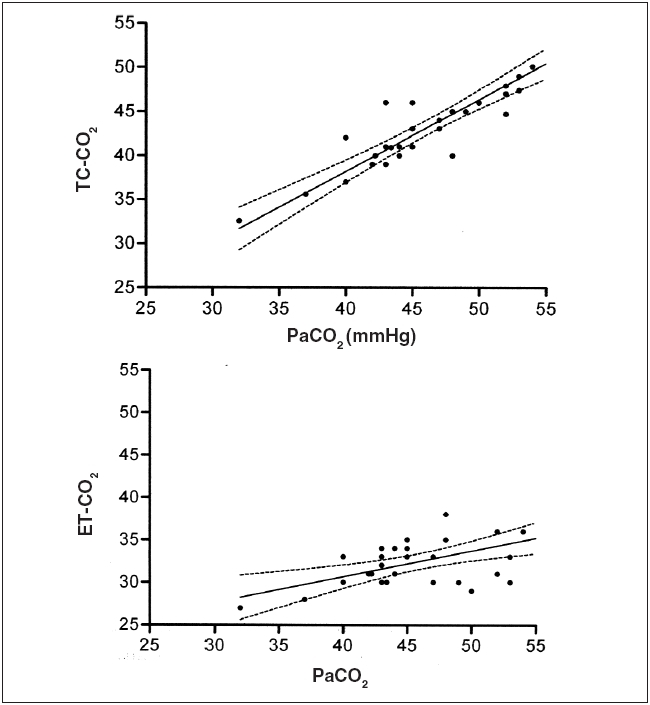
Linear regression analysis during one-lung ventilation of TC-CO_2_ versus PaCO_2_ (above) and ET-CO_2_ vs. PaCO_2_ (below). Analysis of TC-CO_2_ vs. PaCO_2_ revealed r^2^ = 0.7827 and slope = 0.8142 ± 0.0.07965 (95% confidence intervals: 0.6513 to 0.9771). Analysis of ET-CO_2_ vs. PaCO_2_ revealed r^2^ = 0.2989 and slope = 0.3026 ± 0.08605 (95% confidence intervals: 0.1266 to 0.4786)

During OLV, the difference between the TC-CO_2_ and the PaCO_2_ was 3.5 ± 1.7 mmHg with a range of 2 to 8 mmHg (*P* = NS *vs.* TC-CO_2_ to PaCO_2_ difference during TLV). During OLV, the ET-CO_2_ to PaCO_2_ difference was 9.6 ± 3.6 mmHg with a range of 2 to 15 mmHg (*P* = 0.03 *vs.* ET-CO_2_ to PaCO_2_ difference during TLV; and *P*< 0.0001 *vs.* TC-CO_2_ to PaCO_2_ difference during OLV) [Table 1]. In 13 of the 15 patients, the TC-CO_2_ value was closer to the actual PaCO_2_ than the ET-CO_2_ value (*P*= 0.0001). During OLV, linear regression analysis of TC-CO_2_ *vs.* PaCO_2_ resulted in an r^2^ = 0.7827 and a slope = 0.8142 ± 0.07965 (95% confidence intervals: 0.6513 to 0.9771). During OLV, linear regression analysis of ET-CO_2_ *vs.* PaCO_2_ resulted in an r^2^ = 0.2989 and a slope = 0.3026 ± 0.08605 (95% confidence intervals: 0.1266 to 0.4786) [Figure 2]. During OLV, Bland-Altman analysis of TC *vs.* PaCO_2_ revealed a bias of + 2.9 mmHg and a precision of ± 2.5 mmHg and analysis of ET *vs.* PaCO_2_ revealed a bias of −10.5 mmHg and a precision of ± 6.2 mmHg.

No problems occurred with ET-CO_2_ monitoring during the study period. In two patients, the TC-CO_2_ electrode had to be repositioned after the initial placement to obtain an effective value. No blistering, erythema or skin changes were noted on the earlobe following use of the TC-CO_2_ device.

## DISCUSSION

The current study demonstrates that noninvasive monitoring of PaCO_2_ using ET-CO_2_ devices may be inaccurate during minimal access thoracoscopic surgery and OLV in adults. In our cohort of 15 patients, TC-CO_2_ monitoring was more accurate than ET-CO_2_ monitoring (difference of 3.0 ± 1.8 mmHg *vs.* 6.2 ± 4.7 mmHg) during the baseline state using TLV. Although no significant change was noted in the TC-CO_2_ to PaCO_2_ gradient during OLV (3.5 ± 1.7 mmHg), the ET-CO_2_ to PaCO_2_ difference increased to 9.6 ± 3.6 mmHg. In 13 of 15 patients, the TC-CO_2_ was closer to the actual PaCO_2_ than the ET-CO_2_ during OLV.

Several factors may be responsible for discrepancies between ET-CO_2_ and PaCO_2_, including technical issues with the monitor; and patient-related factors, including ventilation-perfusion mismatch, dead space and true shunt.[12,13] Whitesell *et al* demonstrated that patients with underlying lung disease had a significantly greater ET-CO_2_ to PaCO_2_ gradient when compared with patients with normal baseline pulmonary function (3.3 ± 0.6 mmHg versus 0.8 ± 0.3 mmHg).[13] Patient positioning has also been shown to have an impact on the accuracy of ET-CO_2_ monitoring.[2,3] With patients undergoing renal or upper ureteral surgery in the supine position, Pansard *et al* reported that the ET-CO_2_ to PaCO_2_ difference was 4.8 ± 3.9 mmHg 10 min after induction and increased to 7.9 ± 3.5 mmHg (*P*<0.01) 5 min after placement of the patients into the lateral decubitus ‘kidney rest’ position. Similar results were reported by Grenier *et al* in a cohort of patients undergoing neurosurgical procedures in the lateral decubitus position.[3]

In addition to these factors, the significant physiologic alterations induced by OLV can be expected to alter the relationship between ET-CO_2_ and PaCO_2_ values. Ip Yam *et al* evaluated the accuracy of ET-CO_2_ during OLV in a cohort of 22 adults undergoing thoracotomy.[4] During TLV, the ET-CO_2_ to PaCO_2_ difference was 1.3 ± 0.6 kPa (1 kPa = 7.5 mmHg) and it was 1.2 ± 0.7 kPa during OLV. Even if the difference for subsequent ABG analysis was corrected by subtracting the gradient from the first ABG analysis, the ET-CO_2_ to PaCO_2_ difference varied from -1.3 to 1.7 kPa. The authors concluded that the efficacy of ET-CO_2_ monitoring during OLV even when using corrected values remains questionable.

Two previous studies have evaluated noninvasive PCO_2_ monitoring during OLV using both TC-CO_2_ and ET-CO_2_ devices in patients undergoing open thoracotomy,[5,6] while there are no previous reports of using such monitoring in patients undergoing minimal access surgery. Oshibuchi *et al* compared the accuracy of TC-CO_2_ and ET-CO_2_ monitoring in a cohort of 26 adult patients undergoing OLV for open thoracotomy and pneumonectomy. The transcutaneous device (TCM3 transcutaneous CO_2_/oxygen device, Radiometer, Copenhagen, Denmark) was applied to the upper part of the patient's dependent arm. Evaluation of the TC-CO_2_ to PaCO_2_ difference revealed a bias of -0.4 mmHg and a precision of ± 2.5 mmHg during TLV and a bias of 1.4 mmHg and a precision of ± 4.3 mmHg during OLV. Evaluation of the ET-CO_2_ to PaCO_2_ difference revealed a bias of -5.8 mmHg and a precision of ± 4.1 mmHg during TLV and a bias of -7.1 mmHg and a precision of ± 4.6 mmHg during OLV. The authors concluded that TC-CO_2_ monitoring was an accurate means of evaluating PaCO_2_ during OLV. Tobias *et al* used the same transcutaneous device in their study of 15 young adult and pediatric patients (14.1 ± 6.1 years, range - 5 to 28 years) undergoing open thoracotomy.[5] During TLV, the TC-CO_2_ to PaCO_2_ difference was 2.5 ± 0.8 mmHg, while the ET-CO_2_ to PaCO_2_ difference was 3.9 ± 1.6 mmHg (*P* = 0.0049). There was a significant increase in the ET-CO_2_ to PaCO_2_ gradient during OLV (5.8 ± 2.3 mmHg), while no change was noted in the TC-CO_2_ to PaCO_2_ difference (2.7 ± 1.4 mmHg).

The previously reviewed studies of Oshibuchi *et al* and Tobias, along with the data from the current cohort of adult patients, demonstrate the inaccuracy of ET-CO_2_ monitoring during OLV and suggest that TC-CO_2_ monitoring is an effective alternative or adjunct. Until recently, transcutaneous CO_2_ monitoring was used most commonly in the neonatal and occasionally in the pediatric ICU population; however, there is growing experience with its use in adult patients in both the operating room and the ICU setting.[7–9,14–16] These studies have demonstrated that TC-CO_2_ is more accurate than ET-CO_2_ in situations where the continuous monitoring of PaCO_2_ is vital.

TC-CO_2_ monitoring may be of particular benefit when the ventilation-perfusion properties of the respiratory system are altered. ET-CO_2_ measures a sample of gas that contains a mixture of gas exhaled from several areas of the airway and alveoli. Regions with a high ventilation-perfusion ratio (dead space) do not participate in gas exchange and therefore the partial pressure of CO_2_ is low or absent. During exhalation, the gas from regions of dead space mixes with the gas from areas of normal ventilation-perfusion ratios, resulting in dilution of the ET-CO_2_ sample and a widening of the ET-CO_2_ to PaCO_2_ difference. Alternatively, areas of low ventilation-perfusion ratios (shunt) result in ineffective gas exchange and the addition of blood with a high partial pressure of CO_2_ to the arterial circulation contributing to the increased ET-CO_2_ to PaCO_2_ gradient.[17–18]

TC-CO_2_ monitoring avoids the effect of sampling gas that may be subject to ventilation-perfusion mismatch. Transcutaneous monitoring relies on cutaneous respiration of the diffusion of gases across the skin. The transcutaneous monitor measures the CO_2_ that is produced by local tissue metabolism and the CO_2_ released from the blood as it flows through the capillaries near the skin surface. The latter is in direct equilibrium with the capillary CO_2_ which is in equilibrium with the arterial CO_2_. Warming of the skin to 42°C by the sensor increases blood flow and CO_2_ solubility, resulting in an even greater diffusion of CO_2_ into the skin and equilibration with capillary and arterial PCO_2_ values.[19,20] The TC-CO_2_ monitor measures the PCO_2_ at the epidermis by using an infrared sensor, pH electrode or a Clark-type electrode.[21] Unlike ET-CO_2_, which typically underestimates actual CO_2_, the transcutaneous method typically overestimates actual CO_2_ by 5.2-6.4 mmHg due to the increased CO_2_ production from local metabolism induced by heating to 42°C. The currently available TC-CO_2_ devices have an internal correction / calibration factor to correct for the heat-induced changes in CO_2_ production. TC-CO_2_ monitoring requires specific training in calibration, preparation, placement and maintenance of the device. Errors in any one of these steps may give false readings. When compared with ET-CO_2_ monitoring, currently available TC-CO_2_ monitors require a longer preparation time, including a 5-min calibration period and then an additional 5-10 min equilibration time after placement on the patient. Although not an issue with the TC-CO_2_ monitor used in the current study, other TC-CO_2_ monitors may require heating the skin to 44-45°C to ensure accuracy. When this is done, there are occasional reports of superficial burns and skin blistering. Technical and patient-related factors may affect the accuracy of TC-CO_2_ monitoring. Improper membrane placement on the sensor or damage to the membrane may affect its accuracy. Patient factors, including skin thickness, skin edema and hypoperfusion (decreased cardiac output, hypovolemia or vasoconstriction), may also alter the diffusion of CO_2_ to the sensor and result in inaccurate readings.[22–24] As no continuous noninvasive monitor can be expected to be 100% accurate, periodic calibration with an arterial sample may be indicated.

## CONCLUSION

The current study adds to the growing body of knowledge demonstrating the efficacy of TC-CO_2_ monitoring in the adult population. This study is the first to evaluate the use of TC-CO_2_ *vs.* ET-CO_2_ during OLV in minimal access surgery. The continuous monitoring of PaCO_2_ may be particularly important during minimal access surgery. In addition to OLV, CO_2_ insufflation, to facilitate surgical visualization, places patients at a higher risk of hypercarbia than those undergoing open procedures. The combination of increased CO_2_ from systemic absorption of the insufflated CO_2_ with alterations in tidal volume imposed by decreasing minute ventilation during OLV makes accurately and continuously monitoring PaCO_2_ vital during minimal access surgery. TC-CO_2_ monitoring is not meant to replace ET-CO_2_ monitoring. Rather, the devices should be used to complement one another, especially in the OR setting. Although our data further demonstrate that TC-CO_2_ monitoring is more accurate than ET-CO_2_ monitoring, ET-CO_2_ remains the standard of care in the OR to document the intratracheal position of the endotracheal tube, to serve as an additional ventilator disconnect monitor and to provide a capnograph for waveform analysis.
